# Differential effect of obesity on the incidence of retinal vein occlusion with and without diabetes: a Korean nationwide cohort study

**DOI:** 10.1038/s41598-020-67375-x

**Published:** 2020-06-29

**Authors:** Dong Won Paik, Kyungdo Han, Se Woong Kang, Don-Il Ham, Sang Jin Kim, Tae-Young Chung, Dong Hui Lim

**Affiliations:** 1 Department of Ophthalmology, Sungkyunkwan University School of Medicine, Samsung Medical Center, Seoul, Republic of Korea; 2 Department of Statistics and Actuarial Science, Soongsil University, Seoul, Republic of Korea; 3 Department of Clinical Research Design & Evaluation, Samsung Advanced Institute for Health Sciences and Technology, Sungkyunkwan University, Seoul, Republic of Korea

**Keywords:** Vision disorders, Obesity, Lifestyle modification, Risk factors

## Abstract

We aimed to evaluate the association between obesity and the incidence of retinal vein occlusion (RVO) with and without diabetes mellitus (DM).This is a retrospective cohort study using Korean National Health Insurance System data. The participants were 23,061,531 adults older than 20 years who received a health examination at least once between 2009 and 2012, and all patients were observed for RVO development until 2015. We used a multivariate adjusted Cox regression analysis to evaluate the association between RVO and body mass index (BMI) with and without DM. The analysis were evaluated via a hazard ratio (HR) and 95% confidence interval (CI). The age-, sex-, and multivariable-adjusted HRs for RVO were stratified by BMI. This population-based study revealed evidence that obesity has a different effect on the incidence of RVO in the presence and absence of DM.In people with DM, a lower BMI was associated with an increased risk of RVO, and a higher BMI was associated with a lower risk for RVO. In people without DM, the correlation was reversed: a lower BMI was associated with a lower risk for RVO and vice versa.

## Introduction

Retinal vein occlusions (RVOs) are a various group of disorders that all involve impaired venous return from the retinal circulation, sudden onset, and the risk of visual morbidity^1^. RVOs can be classified as central retinal vein occlusions (CRVOs), branch retinal vein occlusions, and hemi-retinal vein occlusions, depending on the site of the obstruction. RVOs are the second most common cause of retinal vascular disease and retinal vascular-related blindness, after diabetic retinopathy^2^. Patients with an RVO are at risk of vision loss from the complications of the interrupted blood flow, including vitreous hemorrhage, optic neuropathy, macular ischemia, macular edema, or even tractional retinal detachment. Additionally, RVOs often follow or are related to cardiovascular disease such as acute myocardial infarction and stroke. According to a previously published report, patients with incident RVO have an increased risk of stroke just after the RVO event^3^.

RVO has many well-known systemic and ophthalmic risk factors. Typical atherosclerosis risk factors are commonly associated with all types of RVO, but vein occlusions can also be secondary to other processes such as compression, inflammation, or vasospasm^4,5^. Systemic diseases such as diabetes mellitus (DM), hyperlipidemia (HLD), and hypertension (HTN) are also strongly related to the progression of RVO^6^. Data from previous studies suggest that 5% of RVO is related to DM, 20% to HLD, and 48% to HTN^7^. Smoking has also been related to RVO^8^. The Diabetes Control and Complications Study and Blue Mountains Study also reported that DM, arteriosclerosis, HLD, and HTN are risk factors for RVO development^9^.

DM is thus a major risk factor for RVO, and it is expected to affect 592 million people in 2035. Asia already contains many people with DM, and DM incidence is increasing at a much faster rate in Asia than in Western countries^10^.

Although obesity is a well-known risk factor for DM and has been suggested as a risk factor for RVO as well, the association of DM and body mass index (BMI) with RVO in previous studies has not been consistent^11^. Moreover, Asians have been reported to have different associations among the percentage of body fat, BMI, and systemic health risks such as cardiovascular disease compared with Europeans and North Americans. In previous studies, body fat percentage (BF %) and certain risk factors have been reported to be elevated at low BMI values^12,13^. Furthermore, BMI could affect the development of diabetic retinopathy (DR) differently in Western and Asian populations^14^. Whereas in Western populations higher BMI correlated with the incidence of DR, in Asian populations, lower BMI was correlated with DR^13,14^. As far as we know, the association between baseline BMI with and without DM and the following risk of RVO has not yet been clarified or studied on a large scale.

Therefore, we inspected the relationship between BMI with and without DM and future RVO occurrence using nationwide health insurance claims data to determine whether the effect of obesity on RVO incidence varies with the presence of DM. Our underlying hypothesis is that in a Korean population, the incidence of RVO could correlate with lower BMI in DM patients, similar to the previous results showing an inverse association between BMI and DR incidence in Asian populations^15^.

## Results

### Baseline characteristics according to BMI and metabolic health status

The baseline characteristics of the study population are presented in Table 1. The average follow-up period was 5.5 years. Participants without DM (NON DM and IFG) are compared to those with DM (NEW DM and MED DM) under the BMI categories of obese (≥ 25 kg/m^2^) and non-obese (< 25 kg/m^2^). The total sample numbers were 20,931,217 without DM and 2,130,314 with DM. Compared with those without DM, those with DM were older, more likely to be men, had higher BMI, glucose, and systolic and diastolic BP, and lower HDL cholesterol, and a greater prevalence of HTN and dyslipidemia.

**Table 1:** Characteristics of subjects according to body mass index and metabolic health status (N = 23,061,531).

BMI	DM (−)^A^			DM (+)^A^			*p *value^b^
	< 25	≥ 25	*p *value^a^	< 25	≥ 25	*p *value^a^	
	(14,566,832)	(6,364,385)		(1,111,657)	(1,018,657)		
Sex (male, %)	6,692,869 (45.95)	3,771,770 (59.26)		661,685 (59.52)	589,333 (57.85)		< 0.0001
Age (years)	45.86 ± 14.34	48.3 ± 13.38	< 0.0001	59.24 ± 12.1	57.02 ± 11.89	< 0.0001	< 0.0001
Hypertension (%)	2,562,603 (17.59)	2,234,717 (35.11)		580,696 (52.24)	669,337 (65.71)		< 0.0001
Dyslipidemia (%)	1,961,712 (13.47)	1,544,915 (24.27)		439,600 (39.54)	492,350 (48.33)		< 0.0001
Smoking (%)			< 0.0001			< 0.0001	< 0.0001
None	9,456,034 (64.91)	3,591,855 (56.44)		623,505 (56.09)	585,743 (57.5)		
Ex-smoker	1,694,466 (11.63)	1,046,149 (16.44)		193,504 (17.41)	193,453 (18.99)		
Current smoker	3,416,332 (23.45)	1,726,381 (27.13)		294,648 (26.51)	239,461 (23.51)		
Drinking (%)			< 0.0001			< 0.0001	< 0.0001
None	7,890,378 (54.17)	3,194,990 (50.2)		663,989 (59.73)	592,826 (58.2)		
1–2 times/week	5,868,448 (40.29)	2,630,839 (41.34)		359,010 (32.3)	333,445 (32.73)		
≥ 3 times/week	808,006 (5.55)	538,556 (8.46)		88,658 (7.98)	92,386 (9.07)		
Exercise (%)	7,148,022 (49.07)	3,304,327 (51.92)		519,315 (46.72)	480,136 (47.13)		< 0.0001
Income (N^A^, %)^c^	3,956,042 (27.16)	1,608,968 (25.28)		306,553 (27.58)	273,738 (26.87)		< 0.0001
Height (cm)	163.24 ± 8.92	164.27 ± 9.74	< 0.0001	161.93 ± 9.02	162.27 ± 9.47	< 0.0001	< 0.0001
Weight (kg)	58.55 ± 8.62	74.03 ± 10.44	< 0.0001	59.39 ± 8.29	73.32 ± 10.41	< 00.0001	< 0.0001
BMI (kg/m^2^)	21.9 ± 2	27.34 ± 2.13	< 0.0001	22.57 ± 1.81	27.76 ± 2.42	< 0.0001	< 0.0001
Waist circumference (cm)	75.78 ± 7.32	87.97 ± 7.01	< 0.0001	80.65 ± 6.61	90.83 ± 7.17	< 0.0001	< 0.0001
Systolic BP (mmHg)	119.23 ± 14.53	126.77 ± 14.5	< 0.0001	127.33 ± 15.97	131.16 ± 15.42	< 0.0001	< 0.0001
Diastolic BP (mmHg)	74.33 ± 9.75	79.09 ± 9.92	< 0.0001	77.56 ± 10.08	80.4 ± 10.1	< 0.0001	< 0.0001
Fasting glucose (mg/dL)	91.58 ± 11.04	95.12 ± 11.61	< 0.0001	146.13 ± 48.06	144.6 ± 43.78	< 0.0001	< 0.0001
Total cholesterol (mg/dL)	191.14 ± 35.41	202.79 ± 36.91	< 0.0001	192.69 ± 41.83	198.28 ± 42.5	< 0.0001	< 0.0001
HDL (mg/dL)	57.62 ± 16.46	51.91 ± 16.11	< 0.0001	52.43 ± 17.78	49.94 ± 16.69	< 0.0001	< 0.0001
Creatinine (mg/dL)	0.95 ± 0.78	0.98 ± 0.69	< 0.0001	0.99 ± 0.72	0.99 ± 0.65	< 0.0001	< 0.0001
eGFR (mL/min)	90.84 ± 39.53	88.12 ± 40.47	< 0.0001	85.22 ± 35.44	84.31 ± 35.98	< 0.0001	< 0.0001

Data are expressed as the mean (SD), %, or geometric mean.

*SD* standard deviation, *BMI* body mass index, *DM* diabetic mellitus, *IFG* impaired fasting glycemia, *BP* blood pressure, *HDL* high density lipoprotein, *eGFR* estimated glomerular filtration rate.

^A^Subjects were categorized as NO DM and with DM groups; the NO DM group included the NON DM (non-diabetic subjects at baseline) and IFG (type of prediabetes, fasting plasma glucose level from 110 mg/dL to 125 mg/dL) groups, and the with DM group comprised the NEW DM (onset of DM who had fasting blood glucose levels ≥ 126 mg/dL at the baseline health examination without previous DM diagnosis) and MED DM (use of insulin or oral hypoglycemia medications from the baseline with diagnosis) groups.

^a^p value—Comparison was performed by student’s t test for continuous variables and Chi-squared test for categorical variables.

^b^p value—Comparison was performed by student’s t test for continuous variables and Chi-squared test for categorical variables between NO DM and DM.

^c^The number of subjects with an income lower than 20% of the total population.

### Risk of RVO occurrence

Table 2 shows the relationships between baseline BMI and RVO occurrence depending on DM and the associations between baseline WC and RVO incidence. In Model 1, RVO incidence was adjusted for age and sex, and Model 2 was adjusted for age, sex, smoking status, alcohol consumption, exercise, history of hypertension, dyslipidemia, and income status. And in Model 3, eGFR (estimated glomerular filtration rate) was adjusted in addition to model 2. Among the participants without DM, the incidence of RVO was significantly higher in obese and overweight subjects than in subjects with BMI < 23 kg/m^2^. On the contrary, among participants with DM, the incidence of RVO in overweight and obese subjects was significantly lower than in subjects with BMI < 23 kg/m^2^. This result was similar for the WC groups: among participants without DM, the incidence of RVO became significantly higher as the WC increased compared with subjects with smaller WC. On the contrary, among participants with DM the incidence of RVO was significantly lower in subjects with a larger WC than in subjects with a smaller WC.

**Table 2:** Age-, sex- and multivariable-adjusted hazard ratios for the occurrence of retinal vein occlusion according to body mass index and waist circumference with and without diabetes.

	N	Event	Total duration	IR^c,d^	aHR (95% CI)		
					Model 1^a^	Model 2^b^	Model 3^c^
**DM (−)**^A^							
Obesity defined by BMI (kg/m^2^)							
< 18.5	895,295	2,497	4,673,037.53	0.53	0.83 (0.79, 0.86)	0.86 (0.83, 0.90)	0.86 (0.83, 0.90)
18.5–23	8,627,337	32,268	46,160,910.19	0.70	Reference	Reference	Reference
23–25	5,044,200	25,434	27,315,651.41	0.93	1.16 (1.14, 1.17)	1.11 (1.09, 1.13)	1.11 (1.09, 1.12)
25–30	5,668,267	32,603	30,603,492.58	1.07	1.31 (1.29, 1.33)	1.20 (1.18, 1.22)	1.20 (1.18, 1.22)
30≤	696,118	3,623	3,670,898.09	0.99	1.44 (1.39, 1.49)	1.25 (1.20, 1.29)	1.24 (1.20, 1.29)
Obesity defined by WC (cm)							
− 80/75	8,268,610	24,081	44,113,403.28	0.55	0.79 (0.78, 0.81)	0.85 (0.83, 0.86)	0.85 (0.83, 0.86)
85/80	4,882,779	22,592	26,401,406.89	0.86	0.93 (0.92, 0.95)	0.96 (0.94, 0.98)	0.96 (0.94, 0.98)
90/85	3,967,665	23,017	21,462,579.04	1.07	Reference	Reference	Reference
95/90	2,269,971	15,156	12,237,272.1	1.24	1.05 (1.03, 1.07)	1.02 (1.01, 1.04)	1.02 (1.00, 1.04)
100/95	1,000,970	7,647	5,357,408.66	1.43	1.12 (1.09, 1.15)	1.07 (1.04, 1.09)	1.07 (1.04, 1.10)
100/95-	541,222	3,932	2,851,919.84	1.38	1.10 (1.06, 1.14)	1.02 (0.99, 1.06)	1.02 (0.99, 1.06)
**DM (+)**^A^							
Obesity defined by BMI (kg/m^2^)							
< 18.5	33,933	522	160,003.99	3.26	1.11 (1.02, 1.22)	1.17 (1.07, 1.28)	1.17 (1.07, 1.28)
18.5–23	539,656	7,760	2,797,323.5	2.77	Reference	Reference	Reference
23–25	538,068	7,335	2,846,090.89	2.58	0.95 (0.92, 0.98)	0.92 (0.89, 0.95)	0.92 (0.89, 0.95)
25–30	860,389	10,575	4,560,390.89	2.32	0.89 (0.86, 0.91)	0.83 (0.81, 0.86)	0.83 (0.81, 0.86)
30≤	158,268	1,602	824,963.41	1.94	0.82 (0.78, 0.87)	0.74 (0.70, 0.79)	0.74 (0.70, 0.78)
Obesity defined by WC (cm)							
− 80/75	328,633	4,172	1,698,168.16	2.46	1.08 (1.04, 1.12)	1.13 (1.09, 1.18)	1.13 (1.09, 1.18)
85/80	438,323	5,596	2,308,755.36	2.42	1.03 (0.99, 1.07)	1.05 (1.01, 1.09)	1.05 (1.01, 1.09)
90/85	532,944	6,920	2,818,487.39	2.46	Reference	Reference	Reference
95/90	418,545	5,570	2,210,572.59	2.52	1 (0.97, 1.04)	0.98 (0.95, 1.02)	0.98 (0.95, 1.02)
100/95	239,365	3,229	1,258,765.5	2.57	0.99 (0.95, 1.03)	0.96 (0.92, 1.01)	0.96 (0.93, 1.01)
100/95-	172,504	2,307	894,023.68	2.58	1.02 (0.97, 1.06)	0.97 (0.92, 1.01)	0.97 (0.92, 1.01)

Data are expressed as the HR (95% confidence interval).

All rates are expressed as the number per 1,000 person-years.

*BMI* body mass index, *WC* waist circumference, *DM* diabetic mellitus, *aHR* adjusted hazard ratio, *CI* confidence interval, *IR* incidence rate, *eGFR* estimated glomerular filtration rate.

^A^Subjects were categorized as NO DM and with DM groups; the NO DM group included the NON DM (non-diabetic subjects at baseline) and IFG (type of prediabetes, fasting plasma glucose level from 110 mg/dL to 125 mg/dL) groups, and the with DM group comprised the NEW DM (onset of DM who had fasting blood glucose levels ≥ 126 mg/dL at baseline health examination without previous DM diagnosis) and MED DM (use of insulin or oral hypoglycemia medications from the baseline with diagnosis) groups.

^a^Model 1: Adjusted for age and sex.

^b^Model 2: Adjusted for Model 1 + smoking, alcohol consumption, exercise, income, hypertension, and dyslipidemia.

^c^Model 3: Adjusted for Model 2 + eGFR.

^d^Incidence rate per 1,000 person-years.

We also analyzed the results by 5-year period to see whether the outcome would change depending on the duration of DM (Supplementary Table S1). Among NON DM and IFG subjects, RVO incidence increased along with the BMI, but in all MED DM subjects, those with a DM duration of less than 5 years and more than 5 years who had been using insulin or oral hypoglycemia medications at baseline, RVO incidence decreased as BMI increased. In NEW DM subjects, no clear trend according to BMI emerged. The inverse association between BMI and RVO (a higher BMI correlating with a lower risk of RVO) was significant in the MED DM subjects, especially those who had DM for more than 5 years.

Likewise, in NON DM, IFG, and NEW DM subjects, RVO incidence mostly increased as the WC increased, whereas in the MED DM subjects, RVO incidence decreased as the WC increased.

**Figure 1:**
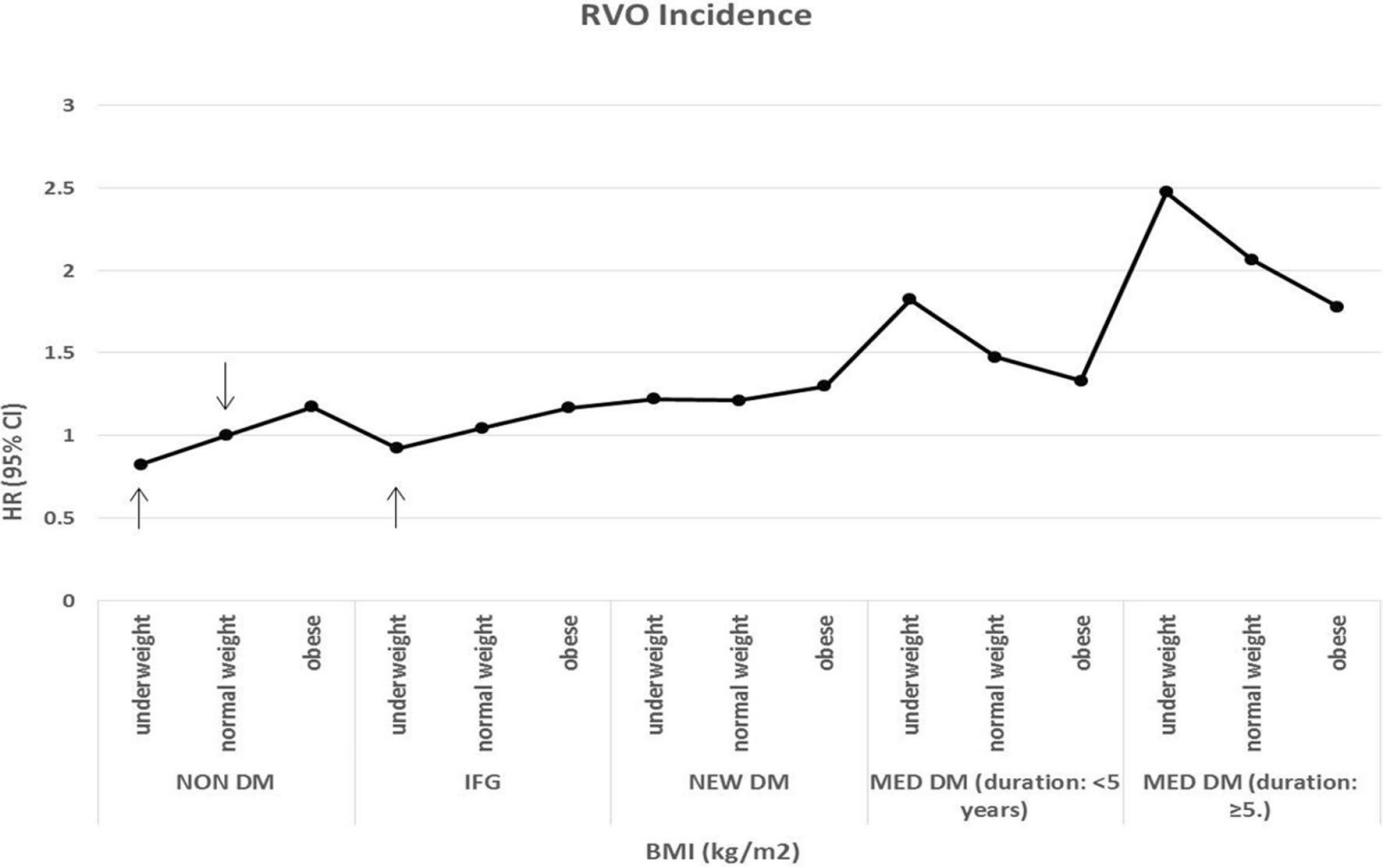
The trend in retinal vein occlusion occurrence according to body mass index and the degree of diabetes. Data are expressed as the HR (95% confidence interval). Adjusted for age, sex, smoking, alcohol consumption, exercise, income, hypertension, and dyslipidemia. ↓Normal weight NON DM participants (reference group). ↑Underweight (BMI < 18.5 kg/m^2^) subjects in the NON DM and IFG groups had lower HR (< 1) than the reference group. *Subjects were categorized as NO DM and with DM groups; the NO DM group included the NON DM (non-diabetic subjects at baseline) and IFG (type of prediabetes, fasting plasma glucose level from 110 to 125 mg/dL) groups, and the with DM group comprised the NEW DM (onset of DM who had fasting blood glucose levels ≥ 126 mg/dL at baseline health examination without previous DM diagnosis) and MED DM (use of insulin or oral hypoglycemia medications from baseline with diagnosis, divided by 5-year durations) groups. *RVO* retinal vein occlusion, *BMI* body mass index, *DM* diabetic mellitus, *HR* hazard ratio, *CI* confidence interval. **Underweight (BMI < 18.5 kg/m^2^), normal weight (18.5 < BMI ≤ 25 kg/m^2^), obesity (BMI > 25 kg/m^2^).

Figure 1 shows the trend of RVO incidence according to BMI (underweight, normal weight, and obese). Compared with the normal weight group without DM (reference group), all subjects in the IFG, NEW DM, and MED DM groups showed significantly higher HRs (95% CI) for RVO, regardless of their BMI, except for underweight (BMI < 18.5 kg/m^2^) subjects in the NON DM and IFG groups. As the duration of DM increased, the HR also increased. But even though a higher degree of DM correlated with higher HRs compared with the reference group, the tendency of RVO incidence showed an opposite trend depending on the degree of diabetes and BMI. The MED DM subjects in Fig. 1 show a significantly inverse association between BMI and RVO (a higher BMI correlates with a lower risk of RVO), especially with DM duration of more than 5 years.

**Figure 2:**
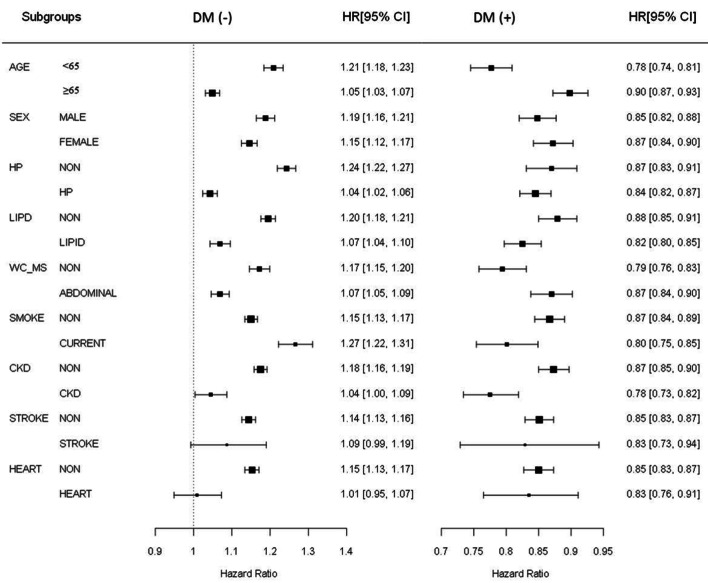
Retinal vein occlusion occurrence for each subgroup of metabolic diseases according to obesity in the NO DM and DM subjects. The HRs (95% CI) were calculated using a Cox proportional hazards model. *WC_MS: WC under metabolic syndrome criteria (abdominal: abdominal obesity). *HR* Hazard ratio, *CI* confidence interval, *HP* hypertension, *LIPD* dyslipidemia, *WC* waist circumference, *MS* metabolic syndrome, *CKD* chronic kidney disease, *HEART* heart failure.

Next, we evaluated the effects of various metabolic disease components on the HRs for RVO incidence according to obesity (Fig. 2). Among the NON DM and IFG subjects, all the subgroups except for stroke and heart failure had an HR value higher than 1, indicating a larger risk for RVO occurrence. Among them, age less than 65 years, non-HTN, non-dyslipidemia, WC below the metabolic syndrome criterion, current smoker, and non-chronic kidney disease were significantly higher in both the NON DM and IFG subjects and did not differ between men and women. On the contrary, among the subjects with DM (NEW DM and MED DM), all the subgroups had an HR value lower than 1, indicating a significantly lower risk for RVO occurrence. Among them, age less than 65 years and having chronic kidney disease were significantly lower in both the NEW DM and MED DM subjects.

## Discussion

In this large-scale, nationwide, long-term follow-up study, we analyzed the incidence of RVO according to BMI, DM, and metabolic status in a Korean population. First, compared with the normal weight group without DM, all subjects in the IFG, NEW DM, and MED DM groups had a significantly higher risk of RVO, regardless of their BMI. Second, although they had a higher risk of RVO compared with the normal weight NON DM subjects, the MED DM subjects showed a significantly inverse association between BMI and RVO incidence (a higher BMI correlated with a lower incidence of RVO), especially with a DM duration of more than 5 years. Third, among subjects without DM (NON DM and IFG), all the subgroups had a higher RVO event rate as they became more obese, irrespective of their metabolic health status. Fourth, among subjects with DM (NEW DM and MED DM), all the subgroups had a lower RVO event rate as they became more obese, irrespective of their metabolic health status.

The pathogenesis of RVO is still not fully understood. It could result from a combination of three systemic changes (Virchow’s triad): (1) blood hypercoagulability, (2) progressive changes in the vessel wall, and (3) hemodynamic changes (venous stasis)^16^. In a recently published paper using data from the Korean NHIS DB from 2013, RVO incidence was higher in women and increased more rapidly with age (incidence doubled after 50 years) among the 754,749 individuals considered. Also, women had a higher age cut-off for RVO than men^17^. Obesity has increased globally and become a major health and social concern, being associated with several major cardiovascular risk factors, including HTN, T2DM, metabolic syndrome, and dyslipidemia^18^.

In previous studies, BMI has been the most commonly used indicator of obesity, with a high BMI correlating with the underlying pathophysiology that can lead to various metabolic diseases and increased mortality. However, that association is not necessarily linear, and recent studies have suggested the existence of an obesity paradox and different ranges of optimal BMI associated with mortality^19^.

In earlier prospective studies, a positive association between increased BMI and DM was consistent, which was well explained as progressive beta cell dysfunction and insulin resistance^11,20^.

Recently, Kim et al. reported that the risk of mortality was lower in patients with moderate obesity (BMI 25 to < 30 kg/m^2^) compared with underweight, normal weight, and overweight members of the general Korean population, supporting the existence of the obesity paradox widely observed among different ethnic groups^21^.

In our study, we found an inverse association between BMI and RVO in DM subjects, especially as the duration of DM became longer. Our results are similar to several cross-sectional studies done in Asia that have shown a protective relationship between DR and being overweight/obese^14^. Because DM is a major risk factor for RVO and DR is a major complication of DM, RVO can be understood through previous DR studies, which showed an inverse association between DR and BMI in DM patients.

The exact mechanism for the inverse correlation between BMI and RVO in the Korean population is unclear. Usually a favorable fat mass/fat-free mass ratio, high potential for optimal treatment, cardiorespiratory fitness, nutritional status, and the cardio-protective metabolic effects of increased body fat have been proposed to explain the protective effect of obesity. Even with the same BMI, Asians' body fat was 3–5% higher than found in Western subjects^22^.

As the duration of DM increases, insulin secretion capacity becomes lower, which leads to a lower BMI compared with participants with a relatively short duration of diabetes, which could help explain the inverse association between BMI and the incidence of RVO, depending on the degree and duration of DM^23^.

In addition, in order to understand the relationship between BMI, RVO, and DM, it was necessary to identify or minimize other factors that could affect each of them through analysis. Among them, nephropathy is an important complication of DM and HTN, which may have different clinical manifestations depending on each cause, and in particular, the weight may decrease or increase depending on renal function, which may affect BMI. Then, the kidney function could be one of the confounding factors in this study. So as shown in Tables 1 and 2, creatinine and eGFR was further analyzed. Especially, eGFR is a measure of kidney function and it could detect kidney disease at an early stage more reliably than the creatinine test alone. Looking at model 3 in Table 2, eGFR was additionally adjusted and analyzed. And like other results, among the participants without DM, the incidence of RVO was significantly higher in obese and overweight subjects than in subjects with BMI < 23 kg/m^2^. And, among participants with DM, the incidence of RVO in overweight and obese subjects was significantly lower than in subjects with BMI < 23 kg/m^2^.

Although our study concluded that a high BMI in DM patients was associated with a reduced risk of developing RVO, care should be taken not to infer that a higher BMI is desirable in all cases. It has been well documented in previous studies that a high BMI correlates with a greater risk of developing both RVO and DM^11^. Even in our study, all subjects in the IFG, NEW DM, and MED DM groups had a significantly higher risk of RVO than the normal weight group without DM. Furthermore, as the degree and duration of DM increased, the incidence of RVO also increased. The inverse association between BMI and RVO incidence was found only in subjects with DM.

The paradox of obesity has provoked controversy about the role of obesity as a major risk factor for death due to HTN, T2DM, metabolic syndrome, cardiovascular disease, and all other causes. The main problems with the obesity paradox are that obesity is regarded as a benign disease and that BMI, not fat distribution, is used to measure total body fat^18^. Athletes can have a BMI similar to that of obese and inactive people, but those BMIs do not have the same meaning. Coutinho and colleagues demonstrated that obesity as defined by the BMI standard was not related to higher mortality rates compared with central obesity^24^. Similarly, previous studies have shown that central obesity can determine cardiovascular risk more accurately than BMI^25^. Therefore, we also used WC, as well as BMI, to determine the effects of central obesity on our outcome more accurately.

The strengths of our study are that it is the first large-scale Korean study to assess how obesity and metabolic health, measured as the degree of diabetes, affect the incidence of RVO using a nationwide data set. For diagnostic accuracy, we used medication prescription data and disease codes, as well as laboratory data from a health examination database that provides a highly representative cohort of the Korean population. We also used not only BMI but also WC to define obesity to accurately reflect body fat and composition. On the other hand, previous studies have shown variations in race and ethnicity in relation to obesity and disease. Although this study was large scale, it cannot be applied globally because it considered only Koreans. Further research might be needed to investigate the diverse effects of obesity on the risk of RVO in Asian versus Western populations.

In conclusion, we showed different risks for RVO incidence according to obesity, DM, metabolic health status, and their interactions in Koreans using a nationwide population-based cohort. In this large cohort, we demonstrated that lower BMI correlated with a higher incidence of RVO in DM subjects, and this inverse association was related to the degree and duration of DM.

## Methods

### Data source

The data evaluated in this study were derived from the national health insurance information database (DB) of the Korean National Health Insurance Service (NHIS). The Korean NHIS is a compulsory public health insurance system that provides general health coverage to the entire population, except for the Medicaid beneficiaries in the lowest income bracket (~ 3% of the population)^26^. Thus, the NHIS DB contains broad information about the health problems of most of the Korean population. The study was approved by the Institutional Review Board of the Samsung Medical Center (SMC IRB number 2018-05-190), and adhered to the tenets of the Declaration of Helsinki. During the national health checkup through NHIS, everyone was asked for their agreement with the consent to use the data. And the Korea National Health Insurance Corporation has allowed authors to use the DB based on the approved IRB.

The Korean NHIS gathers all the information necessary for the reimbursement of each medical service, including patient information, costs gained, disease code, and the place (hospital or clinic) where medical service was received. Patient information available to researchers contains sex, age, residential area (categorized as rural, metropolitan, or city), average monthly insurance premium, and the results of questionnaires (health behavior, medical history), laboratory tests (blood glucose, cholesterol, etc.), and anthropometric measurements (BMI, waist circumference, blood pressure)^27^. Therefore, the Korean NHIS DB has been extensively used in many epidemiological studies^28^. The diagnostic codes used in the DB are based on the Korean Standard Classification of Diseases (KCD), which is similar to the International Classification of Diseases (ICD).

### Study population

**Figure 3:**
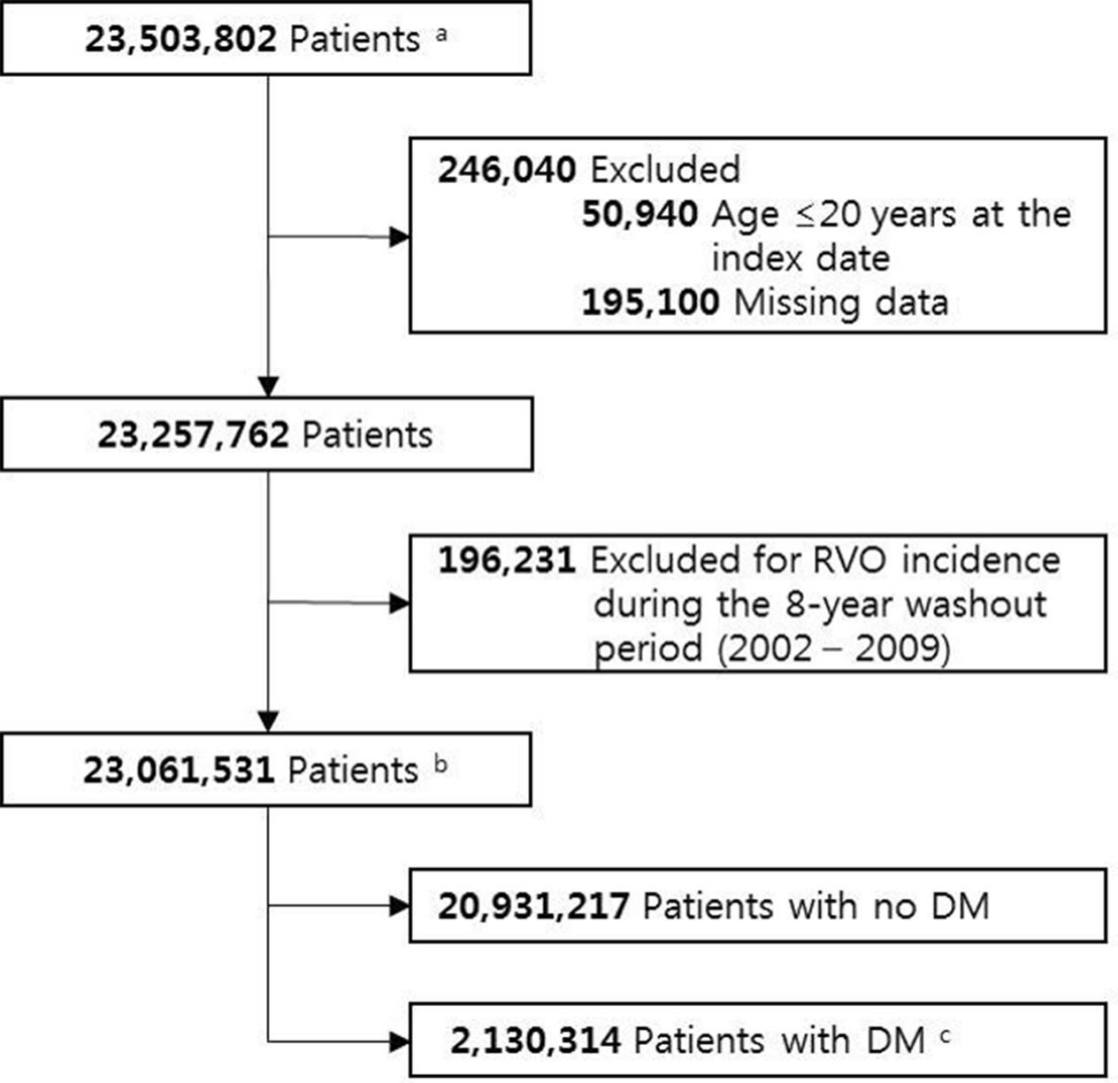
Flow diagram of the process used to identify the study population for the incident retinal vein occlusion cohort. a Patients who participated in the National Health Insurance Service (NHIS) at least once between 2009 and 2012. b Patients diagnosed with retinal vein occlusion (KCD code H34.8 corresponding to ICD-10-clinical modification code 362.35, CRVO, or 362.36, venous tributary (branch) occlusion) during the washout period (2002–2009) were excluded. c Patients diagnosed with Type 2 diabetes mellitus based on ICD-10 codes E11, E12, E13, or E14 and prescribed at least one antidiabetic drug or insulin any time in a given year. *DM* diabetic mellitus.

The NHIS contains health information about 51 million Koreans. We initially enrolled 23,503,802 subjects who participated in the NHIS at least once between 2009 and 2012. Among those subjects, we excluded 50,940 who were younger than 20 years old, 195,100 subjects with missing data, and 196,231 subjects diagnosed with RVO during the washout period (2002–2009). Thus, we included 23,061,531 people older than 20 years who received a health checkup at least once between 1 January 2009 and 31 December 2012; all patients were monitored for RVO development [KCD code H34.8, matching ICD-10-clinical modification code 362.36, venous tributary (branch) occlusion, or 362.35, CRVO] until December 31, 2015. From those subjects, we extracted retrospective data for adult patients with Type 2 DM (T2DM) to compare with non-DM patients. Patients were categorized as having T2DM when they were prescribed at least one anti-diabetic drug or insulin at any time in a given year and recorded at least one service claim with a diagnosis of T2DM (ICD-10 codes E11 to E14) to exclude non-diabetic subjects and those with pre-diabetes^29^. Using guidelines from the national health analysis DB, people with fasting blood glucose levels ≥ 126 mg/dL without a claim for anti-diabetic medication under the ICD-10 code were also considered to have DM. The flow diagram is summarized in Fig. 3.

This study adhered to the Declaration of Helsinki, and the study was reviewed and approved by the Institutional Review Board of Samsung Medical Center (SMC 2018-05-190).

### Measurements and definition of BMI, DM, and other risk factors

BMI was defined as weight in kilograms divided by the square of height in meters (kg/m^2^). In our study, we used the following BMI ranges: underweight (BMI < 18.5 kg/m^2^), normal weight (18.5 ≤ BMI < 23 kg/m^2^), overweight (23 ≤ BMI < 25 kg/m^2^), obese I (25 ≤ BMI < 30 kg/m^2^), and obese II (≥ 30 kg/m^2^), following the Korean Society for the Study of Obesity^30^. Along with BMI, we used waist circumference (WC) to measure obesity, and the cut-off points of WC for abdominal obesity in Koreans were defined as 85 cm for women and 90 cm for men^31^.

For the analysis, patients were classified according to their diabetes status. NEW DM was defined as the onset of DM (fasting blood glucose levels ≥ 126 mg/dL at baseline health examination without a previous DM diagnosis), and MED DM was defined as the use of insulin or oral hypoglycemia medications from baseline with diagnosis. To investigate the effect of DM duration, the MED DM group was divided in two: those who had DM for less than 5 years and those who had DM for more than 5 years at baseline. The IFG group (impaired fasting glycemia) had a type of prediabetes in which their fasting blood glucose level was between 110 mg/dL and 125 mg/dL. The NON DM included subjects who were non-diabetic at baseline.

HTN and dyslipidemia were defined as the use of relevant medications or physician diagnosis or as a systolic blood pressure (SBP) ≥ 140 mmHg or diastolic blood pressure (DBP) ≥ 90 mmHg or total cholesterol ≥ 240 mg/dL.

### Statistical analysis

Data are summarized as mean values with standard deviations (SD) for continuous variables, and numbers with percentages for categorical variables.

Multivariate adjusted Cox regression analyses were used to inspect the hazard ratios (HRs) and 95% confidence interval (CIs) for the associations between RVO and BMI and RVO and WC, both with and without diabetes. For each Cox regression analysis, a p-value for the linear trend across classifications was calculated by announcing the ordinal variable in the model. A p-value < 0.05 was considered statistically significant. Statistical Analysis System software version 9.4 (SAS Inc., Cary, NC, USA) was used for all analyses.

## Supplementary information

Supplementary information.

## References

[1] Laouri M, Chen E, Looman M, Gallagher M (2011). The burden of disease of retinal vein occlusion: Review of the literature. Eye..

[2] Keane PA, Sadda SR (2011). Retinal vein occlusion and macular edema—Critical evaluation of the clinical value of ranibizumab. Clin. Ophthalmol..

[3] Park SJ, Choi NK, Park KH, Woo SJ (2015). Risk of stroke in retinal vein occlusion. Am. Acad. Neurol..

[4] O’Mahoney PR, Wong DT, Ray JG (2008). Retinal vein occlusion and traditional risk factors for atherosclerosis. Arch. Ophthalmol..

[5] Yen YC, Weng SF, Chen HA, Lin YS (2013). Risk of retinal vein occlusion in patients with systemic lupus erythematosus: A population-based cohort study. Br. J. Ophthalmol..

[6] Mohamed Q, McIntosh RL, Saw SM, Wong TY (2007). Interventions for central retinal vein occlusion. An evidencebased systematic review. Ophthalmology.

[7] Ehlers JP, Fekrat S (2011). Retinal vein occlusion: Beyond the acute event. Surv. Ophthalmol..

[8] Klein R, Klein BE, Moss SE, Meuer SM (2000). The epidemiology of retinal vein occlusion: The beaver dam eye study. Trans. Am. Ophthalmol. Soc..

[9] Eye Disease Case-Control StudyGroup (1996). Risk factors for central retinal vein occlusion. Arch. Ophthalmol..

[10] Leasher JL, Bourne RR, Flaxman SR (2016). Global estimates on the number of people blind or visually impaired by diabetic retinopathy: A meta-analysis from 1990 to 2010. Diabetes Care.

[11] Nguyen NT, Nguyen XM, Lane J, Wang P (2011). Relationship between obesity and diabetes in a US adult population: Findings from the National Health and Nutrition Examination Survey, 1999–2006. Obes. Surg..

[12] Consultation WHOE (2004). Appropriate body-mass index for Asian populations and its implications for policy and intervention strategies. Lancet.

[13] Rush EC, Freitas I, Plank LD (2009). Body size, body composition and fat distribution: Comparative analysis of European, Maori, Pacific Island and Asian Indian adults. Br. J. Nutr..

[14] Dirani M, Xie J, Fenwick E (2011). Are obesity and anthropometry risk factors for diabetic retinopathy? The diabetes management project. Investig. Ophthalmol. Vis. Sci..

[15] Chan JCY, Chee ML, Tan NYQ (2018). Differential effect of body mass index on the incidence of diabetes and diabetic retinopathy in two Asian populations. Nutr. Diabetes..

[16] Zhou JQ, Xu L, Wang S (2013). The 10-year incidence and risk factors of retinal vein occlusion: The Beijing eye study. Ophthalmology.

[17] Shin KU, Lee JY, Han KD (2018). Sex-specific age threshold for increased risk of retinal vein occlusion in Koreans. Thromb. Res..

[18] Goyal A, Nimmakayala KR, Zonszein J (2014). Is there a paradox in obesity?. Cardiol. Rev..

[19] Jee SH, Sull JW, Park J (2006). Body-mass index and mortality in Korean men and women. N. Engl. J. Med..

[20] Eckel RH, Kahn SE, Ferrannini E (2011). Obesity and type 2 diabetes: What can be unified and what needs to be individualized?. J. Clin. Endocrinol. Metab..

[21] Kim NH, Lee J, Kim TJ (2015). Body mass index and mortality in the general population and in subjects with chronic disease in Korea: a nationwide cohort study (2002–2010). PLoS ONE.

[22] Deurenberg P, Deurenberg-Yap M, Guricci S (2002). Asians are different from Caucasians and from each other in their body mass index/body fat per centrelationship. Obes. Rev..

[23] Gupta D, Krueger CB, Lastra G (2012). Over-nutrition, obesity and insulin resistance in the development of beta-cell dysfunction. Curr. Diabetes Rev..

[24] Coutinho T, Goel K, de Sa CD (2011). Central obesity and survival in subjects with coronary artery disease. J. Am. Coll. Cardiol..

[25] Lee CM, Huxley RR, Wildman RP (2008). Indices of abdominal obesity are better discreminators of cardiovascular risk factors than BMI: A meta-analysis. J. Clin. Epidemiol..

[26] Song YJ (2009). The South Korean health care system. JMAJ..

[27] Lee H, Cho J, Shin DW (2015). Association of cardiovascular health screening with mortality, clinical outcomes, and health care cost: A nationwide cohort study. Prev. Med..

[28] Chun SH, Cho B, Yang HK (2017). Performance on physical function tests and the risk of fractures and admissions: Findings from a national health screening of 557,648 community-dwelling older adults. Arch. Gerontol. Geriatr..

[29] Ko SH, Kim DJ, Park JH (2016). Trends of antidiabetic drug use in adult type 2 diabetes in Korea in 2002–2013: Nationwide population-based cohort study. Medicine (Baltimore)..

[30] World Health Organization Western Pacific Region (2000). The Asia-Pacific Perspective: Redefining Obesity and Its Treatment.

[31] Lee A, Kim YJ, Oh SW (2018). Cut-off values for visceral fat area identifying Korean adults at risk for metabolic syndrome. Korean J. Fam. Med..

