# Supplementary material for: Differential effect of obesity on the incidence of retinal vein occlusion with and without diabetes: a Korean nationwide cohort study
Source: Sci Rep. 2020 Jun 29;10:10512. doi: 10.1038/s41598-020-67375-x (PMC7324392; doi:10.1038/s41598-020-67375-x)
Supplement: Supplementary file 1 — Supplementary information. [file 41598_2020_67375_MOESM1_ESM.pdf]

Differential effect of obesity on the incidence of retinal vein occlusion with and without diabetes: a Korean nationwide cohort study

Dong Won Paik, MD[1],Kyungdo Han, PhD[3], Se Woong Kang, PhD[1],

Don-Il Ham, PhD[1], Sang Jin Kim, PhD[1], Tae-Young Chung, PhD[1]*,

Dong Hui Lim, PhD[1,2]*

[1]Department of Ophthalmology, Samsung Medical Center, Sungkyunkwan University School of Medicine, Seoul, Republic of Korea.

[2]Department of Preventive Medicine, Graduate School, The Catholic University of Korea, Seoul, Republic of Korea.

[3]Department of Biostatistics, College of Medicine, TheCatholic University of Korea, Seoul, Republic of Korea

*Drs. Dong Hui Lim and Tae-Young Chung contributed equally to this article.

*Correspondence: Dong Hui Lim, PhD (E-mail: ldhlse@gmail.com)

and Tae-Young Chung, PhD (E-mail: tychung@skku.edu)

Representative corresponding author
Dong Hui Lim
Department of Ophthalmology, Samsung Medical Center, Sungkyunkwan University School of Medicine, #81 Irwon-ro, Gangnam-gu, Seoul 06351, South Korea
TEL: 02) 3410-3569, FAX: 02) 3410- 0074
E-mail: ldhlse@gmail.com

Supplementary Table S1. Multivariable-adjusted hazard ratios for occurrence of retinal vein occlusion according to body mass index and waist circumference depending on the degree and duration of diabetes

	HR (95% CI) [a]				
	NON DM	IFG	NEW DM	MED DM (5>Dur)	MED DM (5≤Dur)
Obesity defined by BMI (kg/m^2)					
<18.5	0.853 (0.814,0.894)	0.912 (0.839,0.992)	1.126 (0.913,1.389)	1.24 (1.046,1.47)	1.209 (1.077,1.357)
18.5-23	*Reference*	*Reference*	*Reference*	*Reference*	*Reference*
23-25	1.123 (1.101,1.145)	1.056 (1.024,1.09)	1.073 (0.986,1.169)	0.893 (0.841,0.948)	0.92 (0.882,0.958)
25-30	1.215 (1.192,1.238)	1.148 (1.116,1.181)	1.122 (1.039,1.212)	0.835 (0.79,0.882)	0.831 (0.799,0.865)
30≤	1.259 (1.203,1.317)	1.185 (1.121,1.254)	1.118 (0.983,1.273)	0.797 (0.727,0.873)	0.713 (0.658,0.773)
Obesity defined by WC (cm)					
-80/75	0.842 (0.823, 0.861)	0.877 (0.847, 0.908)	0.918 (0.834, 1.011)	1.139 (1.059, 1.225)	1.161 (1.104, 1.222)
85/80	0.961 (0.94, 0.983)	0.966 (0.935, 0.999)	0.931 (0.852, 1.017)	1.083 (1.015, 1.156)	1.042 (0.995, 1.092)
90/85	*Reference*	*Reference*	*Reference*	*Reference*	*Reference*
95/90	1.027 (1.001, 1.054)	1.02 (0.985, 1.057)	1.021 (0.936, 1.114)	0.99 (0.929, 1.055)	0.984 (0.938, 1.032)
100/95	1.06 (1.026, 1.096)	1.083 (1.038, 1.13)	0.998 (0.898, 1.109)	1.006 (0.935, 1.083)	0.971 (0.917, 1.028)
100/95-	1.021 (0.977, 1.067)	1.03 (0.976, 1.087)	1.147 (1.02, 1.29)	0.976 (0.899, 1.06)	0.963 (0.902, 1.029)

Data are expressed as the HR (95% confidence interval).

a Adjusted for age, sex, smoking, alcohol consumption, exercise, income, hypertension, and dyslipidemia

*Subjects were categorized as NO DM and with DM groups; the NO DM group included the NON DM (non-diabetic subjects at baseline) and IFG (type of prediabetes, fasting plasma glucose level from 110 mg/dL to 125 mg/dL) groups, and the with DM group comprised the NEW DM (onset of DM who had fasting blood glucose levels ≥126 mg/dL at baseline health examination without previous DM diagnosis) and MED DM (use of insulin or oral hypoglycemia medications from the baseline with diagnosis, divided by 5-year durations) groups.

Note: BMI = body mass index, WC = waist circumference, HR = hazard ratio, CI = confidence interval, Dur = duration, DM = diabetic mellitus
